# Differences in *MUC4* Expression in Pancreatic Cancers and Pancreatic Cysts in Egypt

**DOI:** 10.4172/2157-2518.1000312

**Published:** 2018-03-26

**Authors:** Asserewou Etekpo, Ahmad Alghawalby, Marwa Alghawalby, Amr S Soliman, Ahmed Hablas, Baojiang Chen, Surinder Batra, Ghada A Soliman

**Affiliations:** 1 Department of Epidemiology, University of Nebraska Medical Center, College of Public Health, Omaha Nebraska, USA; 2 Department of Radiotherapy, Faculty of Medicine, Mansoura University, Mansoura, Egypt; 3 Department of Community Health and Social Medicine, School of Medicine, City University of New York, New York, USA; 4 The Gharbiah Cancer Society, Tanta, Egypt; 5 Department of Biostatistics, University of Nebraska Medical Center, College of Public Health, Omaha, Nebraska, USA; 6 Department of Biochemistry and Molecular Biology, College of Medicine, University of Nebraska Medical Center, Omaha Nebraska, USA; 7 Department of Environmental, Occupational, and Geospatial Health Sciences, Graduate School of Public Health and Health Policy, City University of New York, New York, USA; 8 Advanced Science Research Center, City University of New York, New York, USA

**Keywords:** Pancreatic cancer, pancreatic cysts, *MUC4*, Egypt

## Abstract

Pancreatic cancer is the fourth cause of cancer deaths in the U.S. with most patients diagnosed at advanced stages followed by short survival. Therefore, biomarkers for early detection are urgently needed. Mucin 4 (*MUC4*) is a mucin protein encoded by the *MUC4* gene and identified in the majority of pancreatic cancers. With increasing clinical identification and diagnosis of pancreatic cysts globally and transformation of some cysts into pancreatic cancer, it is important to evaluate if *MUC4* is expressed in pancreatic cysts.

Immunohistochemistry assays utilizing heat-induced epitope retrieval (HIER) were performed to examine *MUC4* protein expression in 44 paraffin-embedded tissues of pancreatic cancers and 20 pancreatic cysts. All patients were diagnosed and operated upon at the Mansoura University Gastrointestinal Surgery Center in Egypt. Clinical, demographic, and survival information were abstracted from the patients’ medical records. Logistic regression was performed to predict expression of *MUC4* protein in cancer and cysts, by type of cysts.

Pancreatic cyst patients were significantly younger than pancreatic cancer patients (Mean age of 28.7 ± 5.25 *vs.* 54.84 ± 10.60 years) (p=0.0001). Expression of *MUC4* was not different between cancers and pancreatic cysts (p=0.16). However, type of pancreatic cysts was predictive of *MUC4* expression. Mucinous cystic neoplasms and serous cystadenoma cysts showed significantly higher *MUC4* expression than non-specified and pseudocysts (80%, 75%, 25%, and 0% expression for the 4 types of cysts, respectively) (p=0.022).

*MUC4* expression may be associated with certain types of cysts. Follow-up of pancreatic cyst patients who show *MUC4* expression might reveal clues to early detection of pancreatic cancer.

## Introduction

Pancreatic Cancer is one of the short-survival cancers with one-year relative survival rate of approximately 29%, and the five-year rate of about 7% in the U.S. [1,2]. Pancreatic cancer is the 19^th^ most common cancer in Africa [3] and is not one of the common cancers in Egypt. However, the disease is diagnosed at advanced disease stages [4] and is characterized by high mortality [5]. Pancreatic cancer in Egypt also occurs at a relatively young age of diagnosis [6] and is more common in polluted regions [7], and shows variation in mutations by place of residence in relation to pollution levels in the country [8,9].

Mucins are high molecular weight glycoproteins that play a protective function for the epithelial cells under normal physiological conditions. Mucins are also involved in the renewal and differentiation of epithelial tissue and modulation of cell adhesion and cell signaling [10]. Mucins have been associated with various types of cancers based on the alteration in their expression. *MUC4* is membrane-bound mucin that is overexpressed in pancreatic cancer but absent in normal pancreas and chronic pancreatitis [11].

Recent studies have shown *MUC4* expression in 91% of tissues of pancreatic cancer patients [12] and increased expression with advancing stages of pancreatic cancer and poor patient survival [13], but *MUC4* is not expressed in normal pancreas [14]. This finding may imply that mucins play a critical role in pancreatic cancer development. The manifestations of these mucins in pancreatic tumor cells indicate that *MUC4* can be potential biomarker for pancreatic cancer diagnosis and possibly early detection.

Due to absence of early stage tissues and serum samples from pancreatic cancer patients, early detection remains a challenge. Therefore, identifying pre-cancerous lesions and biomarker genes involved in the development of pancreatic cancer could be very relevant to early detection.

Pancreatic cysts are lesions of the pancreas that have been identified more frequently in the U.S. and increasingly so in other countries because of improved diagnostic imaging facilities of Magnetic Resonance Imaging (MRI) and Computed Tomography (CT) [15]. The prevalence of pancreatic cysts from different imaging studies of asymptomatic patients ranged between 1–20% [1,2,6,7,16] and different histopathologic types of cysts are identified [15]. A proportion of those cysts have potentials for malignant transformation and new molecular assays might be helpful for differential diagnosis and assessment of early detection of malignant transformation of pancreatic cysts [17].

Therefore, we conducted this study to compare and contrast *MUC4* expression in pancreatic cancer and pancreatic cysts from Egyptian patients diagnosed and treated at the Mansoura University Gastrointestinal Surgery Center. The study also aimed at exploring the relationship between *MUC4* expression pattern and patients’ demographic, occupational, lifestyle factors.

## Material and Methods

### Study site and patient population

The Gastrointestinal Surgery Center (GSC) is a surgical center of the Mansoura University located in Mansoura city, the 4^th^ largest city in Egypt in the East Nile Delta region. The vast majority of patients who are referred to the center reside in the Dakahleya province. The province is the home of approximately 5.9 million individuals who live in the space area of 3470.0 km^2^ [18]. Faculty of the center perform a full range of diagnostic and management gastrointestinal procedures. Approximately, 40% of the patients are treated free-of-charge, 40% by the national health insurance, and 20% of patients pay the treatment expenses out-of-pocket.

This case-case study included 44 pancreatic cancer and 20 pancreatic cyst patients diagnosed and treated at the GSC. The pancreatic cancer patients were diagnosed and confirmed as ductal adenocarcinoma of the pancreas and were included in our previous publications [8,9]. The 2 groups of patients represented consecutive patients managed at the GSC and all tissues had histopathologic confirmation from paraffin-embedded tissue blocks prepared from the resected lesions. The pancreatic cancer represented 26% of all incident pancreatic cancer patients seen at the GSC hospital during the period of 1998–2004. The rest of the patients, who were diagnosed during the same period but not included in this study, had medical or palliative treatment and no surgical resections or biopsies were performed on them. The pancreatic cyst patients were diagnosed and underwent resection at GSC during a period of 2002 to 2015. Histopathologic confirmation of pancreatic cancer and pancreatic cysts were confirmed by pathologists from the GSC in Egypt and 2 pathologists in the U.S. [M.D. Anderson Cancer Center (SRH) and University of Nebraska Medical Center (AL)]. Demographic, clinical, and risk factor information was obtained from the pancreatic cancer patients by interviewing from our previous study [9], and the clinical and risk factor information was abstracted from the medical records of the pancreatic cyst patients. The information included age, occupation (agricultural, professional and technical or administrative), residence (urban versus rural), smoking and family history of cancer. The study was approved by the IRB committees of the University of Nebraska Medical Center and the GSC in Egypt.

### Immunohistochemistry analysis of *MUC4*

Section of the paraffin-embedded tissues blocks of the pancreatic cancer patients and the pancreatic cysts were tested for *MUC4* by immunohistochemistry utilizing heat-induced epitope retrieval (HIER) as described [13]. The slides were baked at 58 degrees overnight in the oven. The next day, the slides were deparaffinized by washing 3 times (10 min each) in xylene solution. The tissues were rehydrated with decreasing concentrations of 200 proof ethanol (100%, 90%, 80% 70% 50% and 30%) then washed with running water for 5 min. The tissues were incubated for 30 min in 3% hydrogen peroxidase (H_2_0_2_) in methanol to block the endogenous peroxidase. Afterwards, the tissues were blocked in 2.5% horse serum (Cat #: MP-750) from VECTOR for 1 h. Without washing the tissue section, primary antibody *MUC4* (8G7) Cat #: SC 53945 from Santa Cruz was added at the dilution of 1:1000 and was incubated overnight. Normal colon mucosa was used as positive control specimen for *MUC4*. For the negative control, pancreatic cancer tissues and pancreatic cyst tissue were used for immunohistochemistry staining and 1X PBS substituted the primary antibody. The tissues were then washed 3 times for 5 minutes, each with 1X PBST to remove the background. The second antibody Mouse/Rabbit I g Cat# MP-7500 R.TU/Normal horse serum from Vector was added enough to cover the slides. The tissues were incubated at room temperature for 1 h. Detection was performed using DAB kit following the manufacturer’s instructions (cat# SK −4100. Vector, Inc.), followed by hematoxylin staining. The slides were washed for 10 min under running water. The tissues were dehydrated with increasing ethanol concentration (70%, 80%, 90%, and 100%) for 5 min, each followed by 5 minutes of xylene wash. The slides were dried for 15–30 min and the permanent mounting solution was added and covered with glass.

### Data management and statistical analysis

A student’s t test was performed to compare means and standard deviations of continuous variables of the 2 patient groups. These variables were: age and intensity of staining. Fisher exact test was performed to determine the difference in proportions between the 2 patient groups for the following variables: sex, smoking status, rural/urban residence, family history of cancer, and occupation. Two logistic regression models were performed to predict *MUC4* expression. The first model was performed to predict expression of *MUC4* in pancreatic cancer versus pancreatic cysts controlling for age, sex, and smoking status. The second model was performed to predict *MUC4* expression based on the type of cysts, controlling for the same variables of the first logistic regression model. The type of cysts was coded as follows: Type 0 (pseudo cyst and not otherwise specified) and Type 1 (mucinous cystic neoplasms and serous cystadenomas). The intensity of *MUC4* expression was grouped in 2 ways. First, staining was scored from 0 to 3, and 0 intensity was coded as “no expression” while any staining from 0.5 to 3 was coded as “expression”. Second, different codes were given based on the degree of staining as follow: 0.5=“very low”, 1=“low”, 1.5=“less moderate”, 2=“moderate”, 2.5=“high”, and 3=“very high” and analyzed as a continuous variable based on the numerical scores. P-value of less than 0.05 was considered statistically significant. All statistical analyses were conducted using SAS 9.3 (SAS, Cary, NC, USA).

## Results

Comparison of the demographic characteristics of the 2 study groups are presented in Table 1. The descriptive statistical analysis showed that pancreatic cyst patients were younger (Mean age of 28.7 ± 5.25 y) compared to pancreatic cancer patients (Mean age 54.84 ± 10.60 y) (p=0.0001). A statistically significant high proportion of men (56.82%) were among the pancreatic cancer group compared to the cyst group (10.00%) while females represented 43.1% and 90.0%, of the pancreatic cancer and cyst groups, respectively (p=0.0004). There was a significant difference between the pancreatic cancer patients and the cyst patients with respect to smoking status. None of the cyst patients were smokers while 54.6% of cancer patients were smokers (p=0.001). In term of residence, 59.9% of pancreatic cancer patients lived in rural area while 80% of the pancreatic cyst patients lived in rural areas (p=0.2615). Pancreatic cancer patients were more employed in farming than pancreatic patients, 36.59% and 20%, for the 2 groups, respectively (p=0.1892). None of the patients from the 2 groups reported having family history of pancreatic cancer.

*MUC4* protein expression was detected using immunohistochemistry. Normal colon mucosa was used as positive control. Pancreatic cancer tissues without primary antibody added was used a negative control. Tables 2a and 2b show the intensity of expression of *MUC4* by type of cysts. Eighty percent of the mucinous cystic neoplasms stained positive and showed an aberrant expression of *MUC4* protein. *MUC4* expression was observed as well at moderate and less intense staining in 75% of serous cystadenomas. In contrast, only 25% of “not otherwise specified cysts” (NOS) showed low expression of *MUC4* and none of the pseudo cysts expressed *MUC4*, suggesting that *MUC4* expression is up-regulated in mucinous cystic neoplasms and serous cystadenomas. *MUC4* expression was not detected in the pseudo cysts. Age and sex did not show significant relationship to the level of *MUC4* expression.

Tables 3a and 3b present the results of the logistic regression models. Table 3a shows that *MUC4* expression was not predictive of the type of the lesion (pancreatic cancers versus cysts) (p=0.106). After controlling for age, sex, and smoking status, *MUC4* remained unpredictable of the type of lesion (pancreatic cancer and pancreatic cyst) (p=0.733).

The second logistic regression model analysis was performed to predict *MUC4* protein expression based on the type of pancreatic cyst (Table 3a). The result showed that mucinous cystic neoplasms and serous cystadenoma cysts, combined, were 18 times more likely to express *MUC4* protein than the pseudo cyst and the not otherwise specified cysts (p=0.022). After controlling for age and sex, the prediction was 16 times in comparing the 2 respective groups (p=0.041). We did not control for smoking status because none of the cyst patients were smokers.

We analyzed the difference between pancreatic cancer and pancreatic cyst in term of intensity (Table 3c). In the first analysis, we considered intensity as a continuous variable and found that pancreatic cancer tissues stained more but with non-statistically significant difference between pancreatic cancers (1.44 ± 0.92) and pancreatic cysts (1.27 ± 1.30), respectively (p=0.5561). The results of the categorical analysis showed that 80% of pancreatic cancers tissues had some staining compared to 60% of pancreatic cysts (p=0.1008).

## Discussion

Our study revealed the following interesting observations. First, the results showed high level of *MUC4* expression in both pancreatic cancer and pancreatic cyst tissues, with no significant difference in the level of expression between the two groups. We also found that higher expression of *MUC4* was not predictive of pancreatic cancer or cyst status. Second, the study revealed higher expression of *MUC4* in mucinous cystic neoplasms and serous cystadenoma cysts than the expression in pseudo cysts and the not otherwise specified cysts. Mucinous cystic neoplasms and serous cystadenoma cysts were predictive of higher expression of *MUC4*.

The results of this study confirmed findings from previous studies that showed *MUC4* expression in intraductal papillary mucinous neoplasm (IPMN) of the pancreas [19] and pancreatic cancer [20,21] with increased *MUC4* expression in advanced stage of the disease [13]. Chronic pancreatitis could impose higher risk for pancreatic cancer but studies that compared *MUC4* expression in pancreatic cancer and chronic pancreatitis showed *MUC4* expression in 91% of pancreatic cancer but not in chronic pancreatitis tissues [11]. Mucins belong to a family of large O-glycoproteins that serve protection function for epithelial cells against various injuries such as inflammation, bacteria, and viruses under normal physiological conditions [22]. During pancreatic carcinogenesis, gradual expression of *MUC4* has been demonstrated by immunohistochemistry in pancreatic intra-epithelial lesions (PanIN) in the rate of 17% of PanIN1A, 36% of PanIN2, and 85% of PanIN3 [22]. In pancreatic ductal adenocarcinoma, the prevalence of *MUC4* expression reached 83 to 89% of tumors [22].

Some pancreatic cysts have potentials to transform into invasive pancreatic cancer [23–25]. Cyst fluids have been used to diagnose the malignancy of cysts by cytological examination, tumor markers, and cyst fluid viscosity [25]. Tumor marker carcinoembryonic antigen (CEA) has the highest diagnostic accuracy of 79% (sensitivity of 73%, specificity of 84%) for discriminating premalignant mucinous cysts from non-mucinous cysts [26]. Also, *MUC5AC* expression was detected in mucinous but not in other types of cysts [26]. No previous studies have investigated *MUC4* expression in pancreatic cysts and our study is the first investigation of this possible association.

Pancreatic cancer remains as one of the most severe types of cancer with poor prognosis and lack of efficient biomarkers for early detection. The mortality rate from pancreatic cancer almost matches its incidence [27,28] and the disease is always diagnosed at advanced stages, where treatment is no longer effective. The absence of specific biomarkers for early detection explains the late diagnosis. Identifying pre-cancerous lesions and genes involved in the development of pancreatic cancer could be crucial for early detection. Several studies have revealed expression of different *MUC* genes in the development of pancreatic carcinogenesis and the pattern of their expression at different stages of tumor progression [13,28–34].

The strengths of this study include the access to a relatively large number of clinically and histologically well-characterized patients from large gastrointestinal surgery center in Egypt, the histopathologic confirmation of the pancreatic cancer and cyst tissues in both Egypt and the U.S., and the availability of clinical and survival information of patients. A limitation of the study could be the hospital-based nature of the study and the limited generalization of the results to other populations in Egypt.

## Summary

This study showed a statistically significant association between some types of cysts (Mucinous cystic neoplasms and serous cystadenoma cysts) and *MUC4* expression. About 60% of the pancreatic cysts expressed MUC4 protein. This suggests that these patients could be at risk for developing pancreatic cancer and may need to be monitored. There are currently no diagnostic indicators that are consistently reliable, obtainable, and conclusive for diagnosing and risk-stratifying pancreatic cysts. Future studies should focus on setting-up a follow-up cohort study of patients with pancreatic cysts in this population. The follow-up of a cohort could provide clues to early detection of pancreatic cancer and better understanding of the risk of pancreatic cancer for pancreatic cyst patients.

## Figures and Tables

**Table 1:** Demographic and Epidemiologic Characteristics of the Study Population.

Variables	Pancreatic Cancer (44)	Pancreatic cyst (20)	P
	N (%)	N (%)	
Age			
Mean ± SD (years)	54.84 ± 10.60	28.70 ± 5.25	0.0001
Sex			
Male	25 (56.82%)	2 (10.00)	0.0004
Female	19 (43.18%)	18 (90.00)	
Smoking			
Yes	24 (54.55)	0 (0.00)	0.0001
No	20 (45.45)	20 (100.0)	
Residence			
Rural	26 (59.09)	8 (80.00)	0.2615
Urban	18 (40.91)	2 (20.00)	
Occupation			
Farming	15 (36.59)	4 (20.00)	0.1892
Non-farming	26 (63.41)	16 (80.00)	
Family history of cancer			
Yes	0 (0)	0 (0)	N/A
No	44 (100)	20 (100)	

•Values in parentheses indicate percentage values; • N indicates the sample size in each group.

**Table 2a:** MUC4 Protein Expression in Type of Cysts.

Intensity	Type of Cyst					
	Mucinous Cystic neoplas ms (N=10)	Serous cystadenoma (N=4)	Pseudo cyst (N=2)	Not Otherwise Specified (N=4)	Total	P
yes	8	3	0	1	12	0.0057
	80%	75%	0%	25%		
no	2	1	2	3	8	
	20%	25%	100%	75%		

**Table 2b:** MUC4 Protein Expression in Pancreatic Cysts by Immunohistochemistry.

Clinical Data				Expression Level	
Sample	Age	Sex	Type of Cyst	MUC4	Percentage of Cells staining
1	25	F	MCN	++++	80%
2	21	F	SCA	+	Less than 40%
3	27	M	SCA	++	50%
4	24	F	MCN	+++++	50–70%
5	37	M	NOS	-	0%
6	30	F	SCA	+++++	90%
7	28	F	SCA	-	Less than10%
8	24	F	PS	-	0%
9	35	F	MCN	-	0%
10	20	F	MCN	+	Less than 10%
11	22	F	MCN	+++++	50–60%
12	27	F	MCN	++++	60–80%
13	35	F	NOS	-	0%
14	30	F	MCN	++	60–70%
15	24	F	NOS	-	0%
16	32	F	NOS	+	20%
17	31	F	MCN	+++++	80%
18	32	F	MCN	-	10%
19	35	F	PS	-	0%
20	35	F	MCN	+++++	90%

MCN=Mucinous cystic neoplasm; SCA=Serous cystic Adenoma; PS=Pseudocyst; NOS=Not otherwise specified. No Stain (−) +Low level(+); Moderate(++); Very moderate(+++); High(++++); Very high(+++++).

**Table 3:** Logistic regression analysis to predict *MUC4* protein expression in pancreatic cysts and pancreatic cancer, to predict *MUC4* protein based on type of pancreatic cyst, and the protein expression in pancreatic cancer and pancreatic cysts.

Table 3a: Logistic regression analysis to predict MUC4 protein expression in pancreatic cysts and pancreatic cancer		
	OR (95% CI)	p
Unadjusted Model		
Cysts	1	
Cancer	03.8 (0.12–1.22)	0.1063
Adjusted Model		
Cysts	1	
Cancer	0.70 (0.09–5.37)	0.7332
Sex		
Female	1	
Male	0.82 (0.17–3.84)	0.807
Smoking		
Yes	1	
No	0.90 (0.17–4.78)	0.909
Age	0.97 (0.90–1.01)	0.408
Table 3b: Logistic regression to predict MUC4 protein based on type of pancreatic cyst		
	OR (95% CI)	P
Unadjusted group		
Type Cyst 0	1	
Type Cyst 1	18.332 (1.508–222.85)	0.0225
Adjusted Group		
Type Cyst 0	1	
Type Cyst 1	15.92 (1.11–228.24)	0.0416
Sex		
Female	1	
Male	0.509 (0.004–68.24)	0.7869
Age	0.849 (0.65–1.09)	0.2051
Smoking	N/A	N/A

Table 3c: MUC4 Protein Expression in Pancreatic Cancer and Pancreatic Cysts			
Variables	Pancreatic cancer n=44	Pancreatic Cyst n=20	P
Intensity as Continuous			
Mean± SD	1.44 ± 0.92	1.27 ± 1.30	0.5561
Intensity as Categorical			
Yes	35 (79.55)	12 (60.00)	
No	9 (20.45)	8 (40.00)	0.1008
